# Strengthening the Rehabilitation System in Ukraine

**DOI:** 10.3389/phrs.2026.1609220

**Published:** 2026-03-25

**Authors:** Anastasiia Boichuk, Anna Fehlbaum, Kateryna Tymruk-Skoropad, Anna Duchenko, Kaspar Wyss, Helen Prytherch

**Affiliations:** 1 Patients of Ukraine, Kyiv, Ukraine; 2 Swiss Tropical and Public Health Instiute, Allschwil, Switzerland; 3 University of Basel, Basel, Switzerland

**Keywords:** assistive technology, disability, health system, rehabilitation, Ukraine

## Abstract

**Background:**

The full-scale invasion of Ukraine has increased demand for rehabilitation services, exacerbating preexisting deficiencies in the rehabilitation system.

**Analysis:**

This policy brief examines Ukrainian legislation, scientific and grey literature, and findings from stakeholder consultations to reveal persistent marginalization of persons with disabilities (PwD) and limited inclusion in education, employment, and public life. The rehabilitation system and its governance remain fragmented and overly institutional, resulting in poor continuity of care, weak interdisciplinary collaboration, and limited community integration. Resource provision is strained by the war and rising demand for assistive devices. Yet, the influx of humanitarian and development actors presents an opportunity to rebuild and modernize systems and narratives.

**Policy Options:**

We propose (i) establishing an integrated governance and coordination mechanism for rehabilitation services (ii) launching scalable patient pathway models linking hospitals, community-based rehabilitation, and accompanying service providers, and (iii) developing a case management system for smooth assistive device provision.

**Conclusion:**

Improving Ukraine’s rehabilitation ecosystem can advance destigmatization, social reintegration, and a rights-based disability framework, offering lessons for other conflict-affected contexts.

## Background

Ukraine has been in a state of protracted conflict since 2014, which escalated with the full-scale Russian invasion in 2022. This escalation has strained an already underfunded health system. Rehabilitation services were only marginally developed up to 2020 and, prior to that, were largely absent as a recognized component of care. The country now faces a dual burden: longstanding systemic weaknesses, including those unrelated to conflict, have been compounded by acute, war-related disruptions. Rehabilitation needs are multi-faceted, encompassing both physical and psychological dimensions, with trauma occurring on a widespread scale and requiring responses that extend beyond the biomedical sphere.

Ukraine’s rehabilitation policy landscape continues to be shaped by its Soviet legacy, characterized by a highly medicalized approach to disability [1]. This model does not see rehabilitation integrated early into the continuum of care but rather as a separate, subsequent process, frequently relying on institutional settings rather than community-based care. This legacy continues to interfere with efforts to establish evidence-based rehabilitation consistent with the human rights model of disability, where financing is linked to scope and quality of services rather than bed capacity. The absence of an integrated governance framework and coordinated patient pathways adds to the complexity of the system, limiting coherence and continuity across sectors and levels of care [2].

The need for effective rehabilitation services has gained renewed urgency in the wake of Russia’s full invasion. Ukraine faces several pressing health issues—a WHO needs assessment carried out in October 2024 found that 68% of Ukrainians reported their health had declined compared to the period before the war [3]. Moreover, the ongoing war has led to a surge in trauma related needs, including physical injuries and psychological trauma, both of which require early intervention and integration, as well as continuity of rehabilitative care. To illustrate, the Ministry of Health (MoH) estimates that between 2022 and the end of 2024, circa 20,000 patients underwent limb amputations, with the actual number likely being far higher [4]. These evolving and multifaceted pressures compound the challenges faced by a healthcare system already under considerable strain from workforce shortages, displacement, and uneven regional capacity [5, 6].

## Analysis

This policy brief draws on background work undertaken between March and April 2025 to inform the design of the planned Trauma Rehabilitation for Ukraine (TRUE) project. This work consisted of a stakeholder assessment and literature review. Using a four-point Likert scale, stakeholders were mapped and categorized according to their level of interest in improved rehabilitation services and their potential influence on system-level change. Ratings were developed through joint assessment by four co-authors, resulting in the identification of 30 stakeholders across six groups: citizens and civil society; health and rehabilitation service providers; government agencies; training and research institutions; business actors; and development partners and donors.

This was supported by a narrative review of peer reviewed and grey literature, as well as a rapid analysis of Ukrainian legislation. The literature search was conducted across PubMed, WHO IRIS, and Google Scholar, complemented by grey literature including policy reports and project documentation. Search terms combined variations of “rehabilitation,” “disability,” “assistive technology,” “health system,” and “Ukraine.” Sources were included if at least two of the co-authors considered they addressed rehabilitation governance, service delivery, financing, or workforce issues in Ukraine. This was complemented by a legislative and policy analysis focused on current national laws, strategies, and regulatory frameworks governing rehabilitation, social protection, and assistive device (AD) provision.

To validate the findings from the narrative literature review and rapid policy analysis, consultations were held with 42 stakeholders through meetings and workshops clearly explained to have the intention to inform the focus areas of the future TRUE project. These included meetings with representatives from government agencies, professional associations, clinical rehabilitation providers at public health institutions, higher education institutions, patient and veteran groups, and international partners. These consultations provided insights into practical challenges, coordination difficulties, and implementation gaps which underpin the policy options presented in this brief.

The following section outlines key findings on the current state of rehabilitation in Ukraine relating to governance, service delivery and resource provision, to inform a multi-pronged approach towards improvements. Priority actions are needed to: (i) implement standards of care and quality control, including standardized procedures to ensure continuity of care; (ii) reduce system fragmentation from the patient perspective; and (iii) strengthen mechanisms for resource provision. Collectively, these measures lay the foundation for a more equitable and inclusive system, improving both the perceptions and lived experiences of persons with disabilities (PwDs).

### Governance

Ukraine’s rehabilitation governance remains fragmented, with responsibilities dispersed across several ministries and governed by an overlapping regulatory framework. The rehabilitation ecosystem is shaped by more than 2,000 legislative documents, including the Constitution of Ukraine, the State Target Program, Barrier-Free Strategy, the National Action Plan for the Implementation of the Convention on the Rights of Persons with Disabilities, and laws on Social Services, and Social Protection for People with Disabilities [2, 7].

Oversight and implementation are distributed across the Ministries of Health, Social Policy, Education and Science, Economy, Defense, and the Ministry for Communities, Ter- ritories and Infrastructure Development [8]. In recent years, the MoH has emerged as the primary actor driving reform, making notable progress such as by integrating rehabilitation services into the Program of Medical Guarantees (PMG) and advancing efforts to align service delivery with international standards of care. However, the legacy of an earlier, sanatorium-based model persists, as do discrepancies resulting from services provided by other ministries, such as those of Social Policy or Defense, and other actors. Parallel systems often operate with limited coordination and differing standards, resulting in duplication of functions, inefficiencies in resource allocation, and gaps in service continuity for PwD. From the user’s perspective, navigating the system remains complex, as multiple administrative bodies maintain separate eligibility criteria, referral pathways, and funding mechanisms.

### Service Delivery

A functional rehabilitation system includes the presence of clear and coordinated pathways that connect services across levels of care and types of service providers [9]. In Ukraine, rehabilitation is disproportionately focused on inpatient care, despite evidence of the benefits of community-based rehabilitation (CBR) and outpatient services that are more accessible and cost effective [10, 11]. The pathways between care settings are fragmented and poorly co- ordinated, meaning that transition from hospital to outpatient or community services–when these exist—are inconsistent. Additionally, the education of the rehabilitation workforce is not focused enough on inter-professional collaboration across disciplines. The result is that PwDs and their families are frequently left to navigate the system alone, without structured mechanisms for referrals, holistic case management, and follow-up care.

Although national level policy frameworks, such as the 2021 Law “On Rehabilitation in the Healthcare Sector,” recognize the need for integrated and holistic pathways, technical assessments carried out by organizations, including WHO, find that practical implementation has been limited [11]. Past decentralization reforms have also contributed to fragmentation, as governance for service delivery was delegated to oblast authorities [12]. Instead of reforms allowing for the proliferation of local solutions, there has been inconsistent implementation and variation in access to services.

Our consultations with local Civil Society Organizations (CSOs) demonstrated their longstanding efforts to promote and deliver CBR, but international support for scalable CBR solutions remains limited. The national system lacks the mechanisms and coherence to link public, private, NGO, and CSO providers effectively. Thus, the system remains plagued by high drop off rates between levels of care, disruptions to the continuity of rehabilitation, and varying service standards [13].

### Resource Provision

Access to assistive devices (AD)—including prosthetics, orthotics, mobility aids, and communication technologies—has emerged as a critical challenge in Ukraine. This mirrors the global picture where the gap between need and provision is particularly acute in settings that are affected by conflict or resource constrained. Assessments, such as those carried out by the WHO and local CSOs, have highlighted gaps in governance, financing, and workforce capacity in the AD system [14, 15].

Evidence supports the role of high-quality AD in improving functionality, recovery, and social inclusion, as well as facilitating participation in education, employment, and public life [16]. In response, programs such as those run by ProtezHub have trained administrative staff in municipalities in centers for the provision of administrative services provision (CPAS) to be able to provide efficient guidance for citizens. There are also other initiatives and efforts to improve service delivery, notably through specialized institutions in larger cities or donor initiatives such as the War Trauma Rehabilitation Initiative.

Despite these efforts, the overall policy for AD provision remains fragmented at the gov- ernance level [17]. The Ministry of Social Policy (MoSP) is responsible for the administration of publicly funded prosthetic and orthotic (P&O) services, including free provision and main- tenance of devices, even for donated equipment [18]. Each ministry that provides AD (such as the Ministry of Economy via the State Employment Service) limits provision and servic- ing to their mandated field. This means that rehabilitation pathways that include AD are not fully integrated into general rehabilitation pathways, as ministries do not collaborate effectively on provision and follow-up care. Patients in need of ADs are left to navigate a complex system between various agencies, ultimately limiting integration and impacting continuity of care.

## Policy Options

### Establish an Integrated Governance and Coordination Mechanism for Rehabilitation

To address fragmentation and duplication between ministries, the governance of the reha bilitation system must be strengthened through the development and enforcement of unified national standards for the admission, discharge, and follow-up of patients along their rehabilitation journey. These standards shall apply across all levels of care and types of facilities, from acute inpatient rehabilitation to community-based outpatient services. The MoH, as the lead institution for rehabilitation within the health system, further coordinates with other relevant ministries to ensure that service delivery is harmonized and that all actors operate under a consistent framework. Rehabilitation services remain integrated under the PMG, while other ministries focus on complementary mandates, such as social reintegration, vocational training, or veteran support, in collaboration with providers and user organizations, thereby reducing overlap and optimizing the use of resources.

Implementation may be hindered by institutional resistance and regulatory constraints, especially related to relinquishing authority or budgetary control of existing programs. Furthermore, a lack of interoperability between current information systems may also pose a practical challenge. The establishment of a formal coordination structure would require significant regulatory and legal adjustments, alongside a strong political commitment. Overcoming these constraints would enable clearer governance arrangements, leading to more efficient service delivery, improved patient pathways, and thereby a better quality and accessibility of care for PwDs.

### Launch Scalable Patient Pathway Management and Models

We recommend building on the MoH’s initial progress toward CBR by developing and launching an optimal patient pathway model to improve coordination across the rehabilitation sector. This model will link inpatient and outpatient rehabilitation service providers, both public and private, through a unified mechanism for pathway and follow-up management. Initiatives such as TRUE could usefully support national stakeholders by designing these mechanisms and supporting implementation at regional and community level. The objective is to formalize coordination practices that currently operate only informally and to develop a structured and scalable referral system. The model would include establishing local rehabilitation provider networks to enable referrals and information exchange, integrating rehabilitation plans into the national eHealth system to standardize data sharing, to formally embed CBR within the national rehabilitation system.

Effective reform depends on coordination across sectors and levels of government. Ukraine’s decentralization provides room for local innovation but also risks uneven implementation where oblasts lack capacity or political will. Furthermore, while involving a mix of providers is essential to improve access and responsiveness, it also requires a coherent governance framework and quality assurance or risks significant fragmentation. The expected outcomes include reduced patient drop-off between care settings, timelier interventions, and greater consistency in service quality. Stronger interprofessional collaboration, which includes more coordinated education and training of the rehabilitation workforce, would further improve continuity of care. The formalization of mono-professional service provision within such pathways could expand local accessibility and extend rehabilitation coverage to underserved areas. Finally, patient involvement in decision-making would further increase patient empowerment and agency, also contributing to the social rehabilitation process.

### Develop a Case Management System That Accompanies Individuals and Their Families Throughout the Assistive Device Process, Ensuring Coordination and Full Integration

AD prescriptions, both for temporary and permanent use, should be integrated into the national eHealth system to ensure interoperability with the MoSP database and strengthen coordination between medical and social services. This could be operationalized through standardized prescription formats and improved data sharing protocols. The MoH retains responsibility for the provision of temporary assistive devices, supported by a clear and sustainable financial mechanism to guarantee continuity and quality of supply. In addition, comprehensive assessments of assistive device needs shall be embedded as a standard component of rehabilitation at all levels of care, through agreed-upon clinical assessment tools and referral criteria, ensuring consistent integration of services and more equitable access for those in need.

Success can be constrained by legislative gaps which leave ministerial mandates unclear, limited technical capacity and data fragmentation, as well as budgetary constraints. However, with the establishment of this policy, we expect improved coordination between institutions, more efficient resource allocation, and a smoother patient experience across the rehabilitation continuum. By unifying P&O services within formal rehabilitation pathways, the system could deliver timelier interventions, reduce costs associated with delays, and enhance patient satisfaction. In the context of increased demand for P&O services due to conflict-related injuries, this approach offers a strategic window of opportunity to strengthen systems that benefit both veterans and civilians.

## Conclusion

This policy brief highlights three key insights into Ukraine’s rehabilitation landscape: governance remains fragmented, resulting in duplication and inefficiencies; patient pathways are not yet optimized or aligned with best practices across actors; and assistive device provision remains insufficiently integrated into the broader rehabilitation system. To address these gaps, we propose three policy options, as outlined in Table 1: (i) establishing an integrated governance and coordination mechanism for rehabilitation services (ii) launching scalable patient pathway models linking hospitals, community-based rehabilitation, and accompanying service providers, and (iii) developing case-management systems for smooth AD provision.

**TABLE 1:** Overview of recommendations, expected outcomes, and potential barriers of policy issues (Ukraine, 2025).

Policy options	Expected outcomes	Potential barriers
Option one: Establish an integrated governance and coordination mechanism for rehabilitation	Greater coherence and accountability in national rehabilitation system; reduction in duplication of services; greater efficiency in allocation of financial and human resources; appropriate care received by patients; greater navigability of system; improved quality and accessibility of services	Institutional resistance; difficulties relinquishing authority or budgetary control; lack of interoperability between information systems; regulatory and legal adjustments necessary; political commitment necessary; sustained donor and technical support
Option two: Launch scalable patient pathway management and models	Reduced patient drop off rates; timelier interventions; increased consistency in service quality; possibility to improve inter-professional collaboration in education; expansion of local accessibility; improvement in access in previously underserved areas; greater patient involvement and empowerment	Need for high levels of coordination; decentralization may lead to capacity issues; requirement of a coherent governance framework and quality assurance mechanisms; need to develop robust mechanisms for evaluation and scalability
Option three: Develop a case management system that accompanies individuals and their families throughout the AD process, ensuring coordination and full integration of AD provision	Improved coordination between institutions; greater efficiency in resource allocation; improved patient experience across rehabilitation continuum; timelier intervention delivery; reduction of costs associated with delays; improvements in patient satisfaction	Reliance on legislative reforms to clarify mandates; need for interoperable data systems; coordination between donors and national authorities; high potential for duplication; need for strong regulation and quality assurance mechanisms, as well as financial oversight; possible limitations in technical capacity

Together, these measures hold promise for improving the quality and efficiency of rehabilitation services enabling reintegration. Developed collaboratively, and informed by international best practices adapted sensitively to Ukraine’s specific context, we posit that these measures will also have further reaching implications towards the building of a more inclusive society. Rehabilitation system development will help to shift Ukraine away from its Soviet legacy towards a more rights- based, bio psychosocial model concerned with empowerment and participation. The current surge in demand driven by conflict-related needs represents a critical window of opportunity to institutionalize these changes. If seized upon, it can contribute to advancing social reintegration and ultimately creating a more equitable Ukraine.

## References

[1] Health Strategic Advisory Group. National Health Reform Strategy for Ukraine 2015- 2020. Report. Ukraine: Strategic Advisory Group for Health (2014). Available online at: https://healthsag.org.ua/wp-content/uploads/2015/03/Strategiya_Engl_for_inet.pdf (Accessed September 01, 2025).

[2] Golyk V Syvak O Grabljevec K Tederko P Gutenbrunner C Nugraha B . Five Years After Development of the National Disability, Health and Rehabilitation Plan for Ukraine: Achievements and Challenges. J Rehabil Med (2021) 53:jrm00160. 10.2340/16501977-2792 33527144 PMC8814850

[3] World Health Organization. Health Needs Assessment of the Adult Population in Ukraine. Copenhagen: WHO Regional Office for Europe (2024). Available online at: https://iris.who.int/server/api/core/bitstreams/a8c2a1a9-6f01-4526-95ae-931377605535/content (Accessed September 01, 2025).

[4] Ukraine Human Development Update. Report. Washington, DC: World Bank (2024). Available online at: https://documents1.worldbank.org/curated/en/099032824073057091/pdf/P181236-d6feb077-28cd-456c-a506-92390987152a.pdf (Accessed June 30, 2025).

[5] Shkodina AD Chopra H Singh I Ahmad S Boiko DI . Healthcare System Amidst the War in Ukraine. Ann Med Surg (Lond) (2022) 80:104271. 10.1016/j.amsu.2022.104271 35958284 PMC9358411

[6] World Bank, Government of Ukraine, European Union, United Nations. Fourth Rapid Damage and Needs Assessment (RDNA4). Report (2025). Available online at: https://ukraine.un.org/sites/default/files/2025-02/P1801741ca39ec0d81b5371ff73a675a0a8.pdf (Accessed September 28, 2025).

[7] Chopyak V Chemerys O Hdyrya O . The Development of the Rehabilitation System in Ukraine. Proceeding Shevchenko Scientific Soc Med Sci (2024) 76:1–3. 10.25040/ntsh2024.02.01

[8] United NationsGlobal Disability Fund. Situational Analysis on the Rights of Persons with Disabilities in Ukraine: Country Full Report. Report (2025). Available online at: https://globaldisabilityfund.org/new/wp-content/uploads/2025/03/SITAN-Ukraine.pdf?.com (Accessed September 28, 2025).

[9] Sturmberg JP O’Halloran DM Martin CM . Understanding Health System Reform – A Complex Adaptive Systems Perspective. J Eval Clin Pract (2012) 18:202–8. 10.1111/j.1365-2753.2011.01792.x 22221420

[10] Witter S Sheikh K Schleiff M . Learning Health Systems in Low-Income and Middle-Income Countries: Exploring Evidence and Expert Insights. BMJ Glob Health (2022) 7:e008115. 10.1136/bmjgh-2021-008115 36130793 PMC9490579

[11] A Situation Assessment of Assistive Technology in Ukraine. Report. Copenhagen: WHO Regional Office for Europe (2022). Available online at: https://iris.who.int/server/api/core/bitstreams/ef4d142d-07c0-488e-9ae2-cdffed270bb6/content (Accessed September 28, 2025).

[12] Avila C . Implementing Health Financing Policies to Overhaul the Healthcare Delivery system in Ukraine. J Hosp Management Health Policy (2021) 5:7. 10.21037/jhmhp-20-97 (Accessed June 29, 2025).

[13] Treger I Kosto A Vadas D Friedman A Lutsky L Kalichman L . Crafting the Future of Community-Based Medical Rehabilitation: Exploring Optimal Models for Non- Inpatient Rehabilitation Services Through a Narrative Review. Int J Environ Res Public Health (2024) 21:1332. 10.3390/ijerph21101332 39457305 PMC11508180

[14] World Health Organization Regional Office for Europe. Technical Support Mission to Ukraine on Disability, Rehabilitation and Assitive Technology: 10 July-6 August 2022. Report (2023). Available online at: https://iris.who.int/bitstream/handle/10665/366376/WHO-EURO-2023-7099-46865-68337-eng.pdf?sequence=1 (Accessed June 29, 2025).

[15] Lawry LL Korona-Bailey J Hamm TE Maddox J Janvrin M Juman L A Qualitative Assessment of War-Related Rehabilitation Needs and Gaps in Ukraine. J Health Popul Nutr (2025) 44:175. 10.1186/s41043-025-00912-4 40442850 PMC12124016

[16] World Health Organization. Global Report on Assistive Technology. Report (2022). Available online at: https://iris.who.int/bitstream/handle/10665/354357/9789240049451-eng.pdf?sequence=1 (Accessed September 01, 2025).

[17] Petrusha S Bayda L Nayarenko V . Provision with Assistive Technologes. Report. Available online at: https://archive.naiu.org.ua/wp-content/uploads/2024/03/NAIU_AssistiveTechnologiesEN_v01.pdf (Accessed September 28, 2025).

[18] Prosthetic Care. Web Page. Available online at: https://www.msp.gov.ua/en/about/klyuchovi-napryamy/protezuvannya (Accessed September 28, 2025).

